# 

**Published:** 1880-02

**Authors:** 


LUCIEN HOWE, M. D.

### CONTENTS:

| | PAGE. |
|---|---|
| ORIGINAL COMMUNICATIONS: | |
| Cacoëpy in Medicine, by J. W. Keene, M. D., | 277 |
| Prolapsus of the Ovaries, by Darwin Colvin, M. D., | 285 |
| "Tonic Treatment" of Syphilis, by P. W. Van Peyma, M. D., | 290 |
| CLINICAL REPORTS: | |
| Gonorrhœal Conjunctivitis, by Lucien Howe, M. D., | 293 |
| Fibro-Cystic Uterine Tumor, reported by William D. Granger, M. D., | 297 |
| Strictura Recti. Linear Rectotomy—Cure, reported by Herman Mynter, M. D., | 299 |
| TRANSLATIONS: | |
| Extirpation of the Kidney. Translated from the German by Herman Mynter, M. D., | 301 |
| SELECTIONS: | |
| Removal of a Large Fibro-Sarcoma of the Uterus by Abdominal Section under Lister's Method, by L. C. Lane, M. D., | 303 |
| Atropia in Traumatic Tetanus, | 308 |
| Osteo-Myelitis of the Long Bones, | 309 |
| The Seton in Chronic Patellar Bursitis, | 310 |
| Iodide of Starch, | 311 |
| How to Gargle the Naso-Pharynx, | 312 |
| SOCIETY REPORTS: | |
| Buffalo Medical Association, | 312 |
| Annual Meeting of the Medical Society of the County of Erie, | 313 |
| EDITORIAL: | |
| Medical Journalism, | 316 |
| The Metric System, | 318 |
| The Health Officer of New York, | 318 |
| Vivisection, | 319 |
| REVIEWS, | 319 |
| Books and Pamphlets received, | 324 |

